# Self-Reported Measures Have a Stronger Association With Dizziness-Related Handicap Compared With Physical Tests in Persons With Persistent Dizziness

**DOI:** 10.3389/fneur.2022.850986

**Published:** 2022-07-15

**Authors:** Lene Kristiansen, Liv H. Magnussen, Kjersti T. Wilhelmsen, Silje Maeland, Stein Helge G. Nordahl, Anders Hovland, Richard Clendaniel, Eleanor Boyle, Birgit Juul-Kristensen

**Affiliations:** ^1^Department of Health and Function, Faculty of Health and Social Sciences, Western Norway University of Applied Sciences, Bergen, Norway; ^2^Norwegian National Advisory Unit on Vestibular Disorders, Department of Otorhinolaryngol and Head and Neck Surgery, Haukeland University Hospital, Bergen, Norway; ^3^Department of Global Public Health and Primary Care, Faculty of Medicine, University of Bergen, Bergen, Norway; ^4^Research Unit for General Practice in Bergen, The Norwegian Research Center, Bergen, Norway; ^5^Department of Clinical Medicine, University of Bergen, Bergen, Norway; ^6^Department of Clinical Psychology, University of Bergen, Bergen, Norway; ^7^Solli District Psychiatric Centre, Bergen, Norway; ^8^Physical Therapy Division, Department of Orthopaedics and Department of Head and Neck Surgery and Communication Sciences, Duke University School of Medicine, Durham, NC, United States; ^9^Department of Sports Science and Clinical Biomechanics, University of Southern Denmark, Odense, Denmark

**Keywords:** persistent dizziness, Dizziness Handicap Inventory, cross-sectional study, psychological characteristics, physical characteristics, regression analysis

## Abstract

**Background:**

Associations between dizziness-related handicap and a variety of self-reported measures have been reported. However, research regarding associations between dizziness-related handicap and aspects of functioning that includes both physical tests and self-reported measures is scarce.

**Objective:**

The purpose of the study was to describe the variations in signs and symptoms in people with persistent dizziness using physical tests and self-reported outcomes across three severity levels of the Dizziness Handicap Inventory (DHI) and investigate their associations with the DHI.

**Method:**

Participants with persistent dizziness (*n* = 107) were included in this cross-sectional study. The participants underwent (1) physical tests (gait tests, grip strength, body flexibility, and movement-induced dizziness) and completed questionnaires regarding (2) psychological measures (Mobility Inventory of Agoraphobia, Body Sensation Questionnaire, Agoraphobic Cognitions Questionnaire, and Hospital Depression and Anxiety Questionnaire), and (3) fatigue, dizziness severity, and quality of life (Chalders Fatigue Scale, Vertigo Symptom Scale-Short Form, and EQ visual analog scale), in addition to the DHI. Data were presented by descriptive statistics for three DHI severity levels (mild, moderate, and severe). A multiple linear backward regression analysis was conducted for each group of measures in relation to the DHI total score, with additional analyses adjusting for age and sex. Based on these results, significant associations were tested in a final regression model.

**Results:**

With increasing severity levels of DHI, the participants demonstrated worse performance on most of the physical tests (preferred and fast gait velocity, dizziness intensity after head movements), presented with worse scores on the self-reported measures (avoidance behavior, fear of bodily sensation, fear of fear itself, psychological distress, fatigue, dizziness severity, quality of life). After adjusting for age and sex, significant associations were found between total DHI and avoidance behavior, psychological distress, dizziness severity, and quality of life, but not with any of the physical tests, explaining almost 56% of the variance of the DHI total score.

**Conclusion:**

There was a trend toward worse scores on physical tests and self-reported measurements with increasing DHI severity level. The DHI seems to be a valuable tool in relation to several self-reported outcomes; however, several signs and symptoms may not be detected by the DHI, and thus, a combination of outcomes should be utilized when examining patients with persistent dizziness.

## Introduction

Dizziness is a common complaint (1, 2) with 16% reporting dizziness or balance problem in a recent Norwegian survey (3). It is a common feature in acute vestibular diseases where an abrupt start of symptoms, such as dizziness, nauseousness, reduced balance, and visual problems occurs (4). The symptoms usually subside within a couple of weeks; however, ~30% of patients develop persistent symptoms (5, 6). When dizziness persists, it is important to also assess symptoms beyond those defined as vestibular (7). These signs and symptoms include reduced gait velocity (8), postural misalignment (9, 10), increased body sway (11, 12), pain (13), and rigid body movements (14), in addition to anxiety and depression (15–19), avoidance behavior (20), and reduced quality of life (QoL) (21). Fatigue has also been reported in patients with vestibular diagnoses (22). These symptoms may influence everyday life, but to our knowledge, a combination of physical tests and self-reported measures on the perception of dizziness-related handicap has not been investigated extensively.

A commonly used outcome measure related to dizziness is the Dizziness Handicap Inventory (DHI) (23), which was developed to evaluate perceived dizziness-related handicap (24). There seems to be less relationship between diagnostic vestibular function tests and DHI (25, 26), suggesting that dizziness-related handicap may be more associated with the perception of signs and symptoms that have a direct impact on everyday life. Previous studies have found relationships between DHI and functional balance tests involving locomotion (27), slower walking velocity and reduced step and stride lengths (28), and an increasing number of self-reported dizziness triggers (e.g., loud sounds and stress) (29). Severe scores on the DHI have also been associated with poorer psychological outcomes such as anxiety, depression, avoidance behavior, fear of bodily sensations, illness perception, and cognitive responses (5, 30). However, there seems to be a lack of knowledge regarding the relationship between DHI and other outcomes, such as muscle strength, body flexibility, movement-induced dizziness, dizziness-severity, and health-related QoL.

Dizziness Handicap Inventory is measured on a continuous scale (0–100 points). Attempts have been made to establish severity levels of the DHI that links to functional abilities on several occasions (31), but less seems to have been published apart from one study categorizing the DHI into mild (0–30 points), moderate (31–60 points), and severe handicap (61–100 points) (32). Patients with a severe DHI level have shown larger functional impairment on balance confidence and number of falls, compared with patients presenting with mild DHI level scores (32), implying that patients in the severe DHI level may need more clinical attention.

Several studies have reported correlations between DHI and a variety of signs and symptoms. However, there seems to be less scientific evidence examining the relationship between DHI and aspects of function which includes both physical tests and self-reported outcomes. This is an important knowledge in the clinical assessment and treatment of these patients. Therefore, the objectives of this study were to describe both physical tests and self-reported outcomes in people with persistent dizziness in relation to DHI severity levels and to investigate the associations between these outcomes and increasing severity of the DHI total score.

## Method

### Setting

This cross-sectional study used baseline data from a randomized controlled trial (RCT) for treating participants with persistent dizziness (LODIP) (33), and the data are presented according to the STROBE guidelines (34). No sample size calculation was conducted as this study was a part of a larger study and explorative in nature (33). Participants were recruited from primary health care, primarily from general practitioners (GPs), directly, or *via* the general public. Some were also recruited from physiotherapists and ear, nose, and throat specialists (ENTs). Participants attended the Western Norway University of Applied Sciences (HVL) for testing and data were collected from 1 February 2016 to 1 May 2019. Details regarding recruitment and testing procedures have been described elsewhere (33). The LODIP trial was registered in ClinicalTrials.gov (NCT02655575) and approved by the Regional Committee for Medical Research Ethics (2014-00921).

### Participants

A total of 107 participants were included in the LODIP trial. Participants were included if they were within working age (18–70 years old) and presented with perceived dizziness that had started abruptly with symptoms lasting for at least 3 months. In addition, the dizziness symptoms had to be initiated or exacerbated by movement. Exclusion criteria were the following: patient-reported non-vestibular reasons for dizziness, diagnoses with fluctuating vestibular symptoms (e.g., Ménière's disease), plans for/had treatment for benign paroxysmal positional vertigo (BPPV) within 1 month, conditions where fast head movements were contraindicated (e.g., whiplash-associated injuries, osteoporosis of the neck), and severe/terminal pathology (e.g., cancer, psychiatric diagnosis). People were also excluded if they had attended group therapy for dizziness within the past year, if they were unable to attend the testing location, or if they could not understand Norwegian sufficiently.

### Data Collection Procedure

Data collection included physical testing and self-reported questionnaires. The physical testing was conducted according to an established protocol, and three physiotherapists were trained to conduct the testing. A detailed description of the testing procedure has been published earlier (33).

### Variables

**Dizziness Handicap Inventory (DHI)** was used to quantify perceived dizziness-related handicap (24). It consists of 25 items where each item is scored “0” (no), “2” (sometimes), or “4” (yes), summing up to a score between 0 and 100 points. Higher scores represent higher levels of perceived dizziness-related handicap. The DHI scores were categorized into mild (0–30), moderate (31–60), or severe (60–100) dizziness-related handicap (32). It has been translated and validated in several languages, including Norwegian (35–38). The Norwegian version of the DHI has shown a high reliability (38).

#### Physical Tests

**Preferred** (walking at their preferred pace) and **fast** (walking as fast as they could) **gait velocity** was tested by timing the participants as they walked through the middle 6 m of an 8-m pathway. A number of two walking trials were performed for each condition, and the average gait velocity for preferred and fast gait was calculated. Gait velocity assessment has been shown to have high reliability in a vestibular population (39).

The **grip strength** test was used as an indicator of overall muscle strength (40). The test has also shown to inform regarding muscle mass, physical function, and health status, as well as to predict future physical function and health across various clinical populations (40). Maximal grip strength of the dominant hand was used as an indication of general muscle health (40) and was measured using a dynamometer (Mie Medical Systems myometer). The average strength (in kg) from two trials of the dominant hand was calculated. The grip strength test testing has shown to have a high reliability and validity in healthy individuals as well as in various patient populations (41).

A total of four elements (lumbosacral flexion, head-nod flexion, shoulder retraction, and elbow-drop) from the movement domain of the **Global Physiotherapy Examination (GPE**) (10, 14) were examined to investigate body flexibility. Each element was scored on a predefined ordinal scale with 15 levels between “−2.3” (hypotonus) and “+2.3” (hypertonus), with “0” representing the normal standard. The absolute values of the positive and negative scores were added to create a sum score, and the median scores were presented. The GPE body flexibility domain has been reported to be reliable and valid in individuals with musculoskeletal pain (42).

Dizziness severity after head movements was tested by a **head movement-induced dizziness test (NRS_dizziness**). The test involved active head oscillations at 1 Hz and guided by a metronome, for 1 min. The perceived intensity of dizziness after head movements was scored on a Numeric Rating Scale (NRS) from “0” (no dizziness) to “10” (as bad as it can be). The NRS has shown acceptable psychometric properties in patients with chronic pain (43).

#### Psychological Measures

Avoidance behavior when being alone was assessed using the **Mobility Inventory of Agoraphobia, alone (MI-A)**. The MI-A contains 27 items, each rated from 1 (never avoid) to 5 (always avoid), and higher scores indicate greater avoidance behavior (44). The **Body Sensation Questionnaire (BSQ)** was used to evaluate fear of bodily sensations associated with panic and anxiety (45). It contains 18 items, each scoring from 1 (not at all frightened) to 5 (extremely frightened), and higher scores imply greater fear of somatic sensations. The **Agoraphobic Cognitions Questionnaire (ACQ)** was used to measure fear (45). It contains 14 items, each scoring from 1 (thought never occurs) to 5 (thought always occurs), and higher scores indicate greater levels of fear of physical, social, and mental consequences related to the symptoms of anxiety. All of these (MI-A, BSQ, and ACQ) have shown acceptable reliability and internal consistency in a population with anxiety (45). In addition, psychological distress was evaluated using the **Hospital Anxiety and Depression Scale (HADS)** (46). The HADS contains 14 items, each rated from 0 (not present) to 3 (considerably present). The sum score (0–42) is reported, and higher scores indicate higher levels of psychological distress. The scale has shown good internal consistency and acceptable validity in people with dizziness (47).

#### Fatigue, Dizziness Severity, and QoL Measures

To evaluate perceived fatigue, the participants completed the **Chalder Fatigue Questionnaire (CFQ)** (48). The score of the 11 items, with each item scoring from 0 (better than usual) to 3 (much worse than usual), is summed, and higher scores indicate more fatigue. The **Vertigo Symptom Scale-Short Form (VSS-SF)** measures the perceived severity of dizziness symptoms during the past month (49). The questionnaire is a 15-item scale, and each item is scored between 0 (never) and 4 (very often). The sum score (0–60) is reported, and a higher score indicates greater symptom severity. The Norwegian version of VSS-SF has shown good reliability and construct validity (50). To evaluate the quality of life (QoL), the participants completed the **EQ-VAS** section of the **EQ5D-5L** (a generic quality of life measure) (51, 52). This section contains a visual analog scale scoring from 0 to 100, where higher scores indicate better health-related QoL. The **EQ-VAS** has shown good reliability in patients with rheumatoid arthritis (53).

### Statistical Methods

Continuous data were checked for normality using Shapiro–Wilk tests and QQ plots. Demographic data (age, sex, duration of dizziness, educational level, and work situation) were collected and presented for the total population, as well as for the three dizziness-related handicap levels (described above). Depending on the nature of the measure, the data were presented using means, medians, or proportions and their respective 95% CI around the point estimate. Due to the number of measures investigated, they were split into three groups for all the analyses. Group 1 included measures collected during testing, termed “Physical tests,” Group 2 was termed psychological measures and included measures related to anxiety, avoidance behavior, and psychological distress, whereas Group 3 comprised comprise other relevant self-reported measures (fatigue, dizziness severity, and QoL) which were also believed to have an impact on DHI scores. The measures were presented according to three groups of outcomes in relation to the severity level of the DHI. Possible differences between the DHI severity levels in each of the measures were investigated by comparing means/ medians in relation to CI in the different measures.

**Figure 1:**
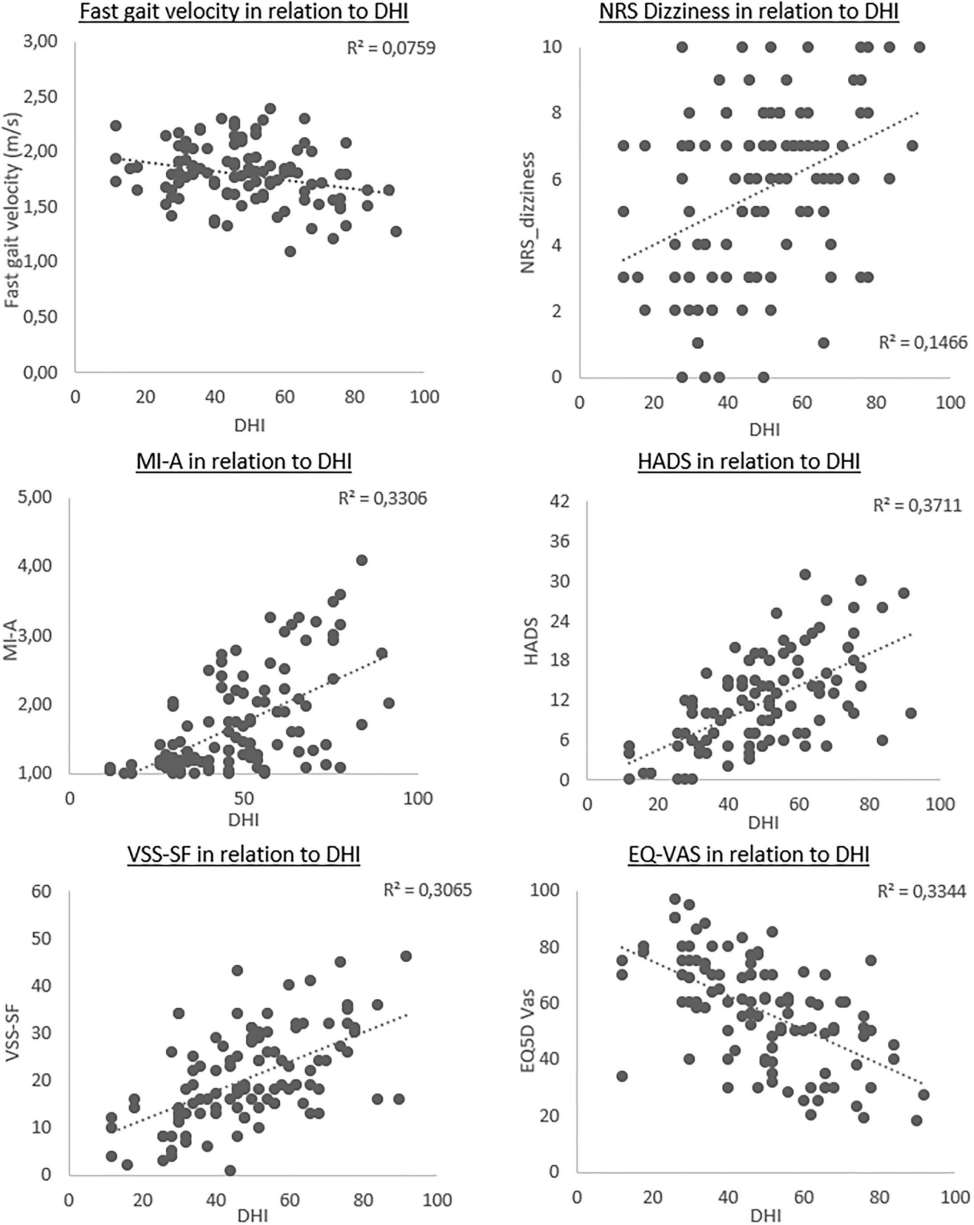
Scatterplots of the outcomes significantly associated with the total Dizziness Handicap Inventory (DHI) score, in particicpants with persistent dizziness. Each plot presents the strength of the linear relationship, with DHI scores along *x*-axis and the different outcome measures on the *y*-axis. The dotted line represents the unadjusted regression coefficient for each outcome.

All the assumptions for linear regressions were met. Backward multiple linear regression analyses were used to test for associations between DHI total score and the measures within each group. Tests for assumptions were conducted to investigate for multicollinearity and normality of residuals. The regression analyses for each group were calculated as one model, including all the measures in each group, which resulted in three separate backward regression models. Regression analyses were afterward adjusted for age and sex. A positive beta coefficient implied a positive association with the DHI, whereas a negative beta coefficient indicated a negative association. The significant measures in each of the unadjusted models were presented in scatterplots in relation to DHI total score (Figure 1). Finally, a regression model including only the measures with *p*-values below 0.10 from each of the three models was conducted, with similar adjustments as conducted for the separate blocks.

The statistical program IBM SPSS Statistics 26 was used for analysis, and *p*-values <0.05 were considered significant.

## Results

A total of 107 participants were included, with a mean age of 49 years (20–70 years), most of the participants were women (76%), and 50% of the participants reported a dizziness duration of at least 2 years (Table 1). The mean DHI score was 49 points (Table 2). Based on the DHI scores, 18% of the participants were classified as having mild dizziness-related handicap, 56% had moderate dizziness-related handicap, and 26% had severe dizziness-related handicap. Participants in the mild category tended to be older (mean age = 54 years) with a higher percentage of women (84%). A similar proportion of participants in the mild and moderate categories experienced dizziness for more than 24 months (53%), but almost two times as many in the moderate category (41%) were currently on sick leave compared with those in the mild category (21%), slightly over a third of the participants in the severe category presented with dizziness for more than 24 months; and 61% of these reported being on sick leave.

**Table 1:** Demographic data among participants with persistent dizziness, presented for the total population and categorized into three levels of severity of the Dizziness Handicap Inventory (DHI).

			**DHI category**	
	**Total population**	**Mild (0–30 points)**	**Moderate (31–60 points)**	**Severe (61–100 points)**
	**107)**	**(*n* = 19, 18%)**	**(*n* = 60, 56%)**	**(*n* = 28, 26%)**
Mean age, years (95% CI)	49 (46; 51)	54 (48; 61)	47 (43; 50)	49 (45;, 54)
Females, % (95% CI)	76 (0.66; 0.83)	84 (0.60; 0.97)	73 (0.60; 0.84)	75 (0.55; 0.89)
Median dizziness duration, months (95% CI)	23^a^ (17; 40)	36^b^ (17; 100)	33^c^ (14; 74)	18^d^ (6; 21)
24+ months, % (95% CI)	50 (0.49; 0.51)	53 (0.28; 0.77)	53 (0.39; 0.66)	39 (0.20; 0.61)
**Educational level**	
High school or below, % (95% CI)	31^e^ (0.22; 0.41)	21 (0.06; 0.46)	32^f^ (0.21; 0.46)	35^g^ (0.17; 0.56)
**Work situation**	
Working, % (95% CI)	41^h^ (0.31; 0.51)	58 (0.33; 0.80)	42^f^ (0.30; 0.56)	25 (0.11; 0.45)
Sick leave or incapacity, % (95% CI)	42 (0.33; 0.52)	21 (0.06; 0.46)	41^f^ (0.28; 0.54)	61 (0.41; 0.78)
Other, % (95% CI)	17 (0.10; 0.26)	21 (0.06; 0.46)	17^f^ (0.84; 0.29)	14 (0.04; 0.33)

*DHI, Dizziness Handicap Inventory; CI, Confidence Interval*.

a *n = 95*,

b *n = 17*,

c *n = 55*,

d *n = 23*,

e *n = 104*,

f *n = 59*,

g *n = 26*,

h *n = 106. The term “other” includes students, persons staying at home, retired, and other*.

**Table 2:** Physical test (Group 1) and self-reported psychological measures (Group 2), and fatigue, dizziness severity and quality of life (Group 3) among participants with persistent dizziness, presented for the total population, and categorized into three levels of severity of the Dizziness Handicap Inventory (DHI).

			**DHI categories**					
	**Total population**		**Mild (0–30 points)**		**Moderate (31–60 points)**		**Severe (61–100 points)**	
	**Mean**	**(95% CI)**	**Mean**	**(95% CI)**	**Mean**	**(95% CI)**	**Mean**	**(95% CI)**
DHI	49	(45.3; 52.4)	24	(20.7; 27.8)	47	(44.5; 48.9)	72	(68.6; 75.6)
**Physical tests (Group 1)**	
Preferred gait velocity (m/s)	1.18	(1.10; 1.18)	1.22	(1.12; 1.32)	1.20	(1.15; 1.24)	1.11	(1.01; 1.20)
Fast gait velocity (m/s)	1.79	(1.74; 1.84)	1.80	(1.69; 1.91)	1.85	(1.78; 1.91)	1.66	(1.55; 1.77)
Grip strength (kg)^*^	25.4	(24.2; 27.0)	24.9	(22.2; 27.3)	26.2	(24.0; 28.4)	24.3	(21.7; 29.3)
GPE movement	3.80	(3.47; 4.13)	3.40	(2.65; 4.15)	3.78	(3.33; 4.23)	4.13	(3.44; 4.93)
NRS_dizziness^*^	6.0	(5.0; 7.0)	5.5	(3.0; 7.0)	6.0	(5.0; 7.0)	7.0	(6.0; 8.0)
**Psychological measures (Group 2)**	
MI-A^*^	1.41	(1.30; 1.70)	1.11	(1.04; 1.26)	1.39	(1.26; 1.76)	2.30	(1.70; 3.00)
BSQ^*^	1.70	(1.50; 1.82)	1.35	(1.12; 1.71)	1.59	(1.47; 1.82)	2.06	(1.76; 2.24)
ACQ^*^	1.30	(1.26; 1.42)	1.16	(1.11; 1.26)	1.37	(1.26; 1.42)	1.66	(1.32; 2.06)
HADS^*^	11.0	(9.0; 12.0)	5.0	(1.0; 7.0)	10.5	(9.0; 13.0)	15.5	(13.0; 22.0)
**Fatigue, dizziness severity, QoL (Group3)**	
CFQ^*^	18.0	(16.0; 20.0)	13.0	(11.0; 17.0)	18.5	(16.0; 21.0)	24.0	(18.0; 27.0)
VSS-SF	20.58	(18.66; 22.49)	12.53	(7.14–18.43)	20.22	(18.47; 22.76)	27.04	(23.39; 31.25)
EQ-VAS (%)	56.73	(53.52; 60.79)	73.22	(67.38–85.91)	59.05	(54.36; 62.76)	42.82	(36.69; 48.11)

* *Indicates median score. DHI, Dizziness Handicap Inventory; CI, confidence interval; m/s, meters per second; kgs, kilograms; GPE, Global Physiotherapy Examination; NRS_dizziness, head movement-induced dizziness; MI-A, Mobility Inventory of Agoraphobia-Alone; BSQ, Body Sensation Questionnaire; ACQ, Agoraphobic Cognitions Questionnaire; HADS, Hospital Anxiety and Depression Scale; QoL, quality of life; CFQ, Chalders Fatigue Questionnaire; VSS-SF, Vertigo Symptom Scale-Short Form*.

Participants presented with poorer scores in all groups of measures with increasing severity levels of DHI (Table 2). In Group 1, there was a tendency of higher preferred and fast gait velocities for those in the mild and moderate DHI categories compared with the severe category, and the participants in the mild DHI severity level demonstrated a trend toward lower NRS_dizziness scores compared with the severe group. The Group 2 measures all presented with low scores in the mild DHI severity level, which increased with DHI severity levels. The measures in Group 3 showed a similar trend of worsening symptoms with increasing severity levels of the DHI (increasing scores in CFQ and VSS-SF, decreasing scores on the EQ-VAS).

A total of three separate backward stepping multivariable models, with additional analyses including age and sex, were fitted to determine which measures within each group were associated with DHI (Table 3). After adjusting for age and sex, we found a significantly negative association between fast gait velocity and the DHI, and a significantly positive association between head movement-induced dizziness and the DHI (Model 4). For the measures in Group 2, the adjusted model showed significantly positive associations between both the MI-A and HADS scores and the DHI (Model 5). In Group 3, there was a significantly positive association between the VSS-SF scores and the DHI, and a significantly negative association between the EQ-VAS and the DHI after including age and sex in the model (Model 6). Model 4 could explain 13% of the variance in the DHI scores, whereas Models 5 and 6 explained 43 and 41% of the variance.

**Table 3:** Associations between total DHI score and measures in the physical (Models 1 & 4), psychological (Models 2 & 5), and “other” domains (Models 3 & 6) in participants with persistent dizziness.

	**Models**				**Models with covarites (Age, sex)**			
	**β**	**95% CI**	***p***	***Adjusted R* ^2^**	**β**	**95% CI**	***P***	***Adjusted R* ^2^**
**Physical tests**	**Model 1**			**0.15**	**Model 4**			**0.13**
**Preferred gait velocity (m/s)**	
Fast gait velocity (m/s)	−12.69	−25.09; −0.29	0.045		−16.55	−29.95; −3.15	0.016	
Grip strength (kg)								
GPE movement								
NRS_dizziness	2.14	0.88; 3.40	0.001		2.04	0.79; 3.29	0.002	
**Psychological questionnaires**	**Model 2**			**0.44**	**Model 5**			**0.43**
MI-A	8.21	3.83; 12.59	<0.001		8.47	4.07:12.87	<0.001	
HADS	1.05	0.60; 1.50	<0.001		1.04	0.58; 1.50	<0.001	
BSQ								
ACQ								
**Other questionnaires**	**Model 3**			**0.44**	**Model 6**			**0.41**
CFQ								
VSS-SF	0.66	0.37; 0.95	<0.001		0.65	0.34; 0.96	<0.001	
EQ-VAS	−0.40	−0.55; −0.25	<0.001		−0.42	−0.57; −0.26	<0.001	

*Shaded outcomes removed from models due to p-values above 0.100. β, beta coefficient; CI, confidence interval; p, p-value; GPE, Global Physiotherapy Examination; NRS_dizziness, head movement-induced dizziness; MI-A, Mobility Inventory of Agoraphobia-Alone; HADS, Hospital Anxiety and Depression Scale; BSQ, Body Sensation Questionnaire; ACQ, Agoraphobic Cognitions Questionnaire; CFQ, Chalders Fatigue Questionnaire; VSS-SF, Vertigo Symptom Scale-Short Form; EQ-VAS, self-rated quality of life*.

The independent variables associated with changes in DHI scores are presented as scatterplots (Figure 1). The plots showed a small negative association between DHI and fast gait, whereas NRS_dizziness presented with a larger variety of scores, with a small positive association. The MI-A, HADS, and VSS-SF all had a clear positive association, whereas the EQ5D-Vas illustrated a negative association with the DHI.

The independent variables in the final model (Model 7) could explain 58% of the variance (Table 4). After adjusting for age and sex, the MI-A, HADS, VSS-SF, and EQ-VAS (Model 8) remained significantly associated, and together, they explained 56% of the variance in the DHI scores.

**Table 4:** Association between the significant outcomes from models 4–6 and the total Dizziness Handicap Inventory (DHI) score, in participants with persistent dizziness.

	**Model 7**				**Model 8 (with covariates age, sex)**			
	**β**	**95% CI**	***p***	***Adjusted R* ^2^**	**β**	**95% CI**	***p***	***Adjusted R* ^2^**
				**0.58**				**0.56**
Fast gait velocity (m/s)	−7.59	−16.92; 1.74	0.110		−6 to 69	−17.21; 3.83	0.209	
NRS_dizziness	0.35	−0.63; 1.33	0.483		0.33	−0.67; 1.32	0.518	
MI-A	6.34	2.21; 10.46	0.003		6.71	2.45; 10.98	0.002	
HADS	0.58	0.13; 1.03	0.012		0.60	0.14; 1.06	0.011	
VSS-SF	0.50	0.23; 0.77	<0.001		0.51	0.22; 0.79	<0.001	
EQ-VAS	−0.21	−0.36; −0.06	0.008		−0.20	−0.36; −0.04	0.015	

*Shaded outcomes; p-values above 0.100. β, beta coefficient; CI, confidence interval; p, p-value; NRS_dz, head movement-induced dizziness; MI-A, Mobility Inventory of Agoraphobia- Alone; HADS, Hospital Anxiety and Depression Scale; VSS-SF, Vertigo Symptom Scale-Short Form*.

## Discussion

There was a clear trend toward worse scores for the different outcomes in each of the DHI levels. In Group 1, gait velocity and head movement-induced dizziness (NRS_dizziness) became poorer with increasing severity level of the DHI. This also applied to avoidance behavior (MI-A), fear of bodily sensations (BSQ), fear itself (ACQ), and psychological distress (HADS) in Group 2, and fatigue (CFQ), dizziness severity (VSS-SF), and health-related QoL (EQ-VAS) in Group 3. In the adjusted multiple regression analyses, significant associations were found between increasing DHI severity and two measures within each of the three groups; fast gait velocity and NRS_dizziness in Group 1, MI-A and HADS in Group 2, and VSS-SF and EQ-VAS in Group 3. In the final adjusted combined model, only the MI-A, HADS, VSS-SF, and EQ-VAS remained significantly associated with increasing DHI severity, explaining 56% of the variance in the DHI score.

The participants scoring in the severe DHI category walked slower than the individuals in both the mild and moderate categories, at both preferred and fast velocities. The current results are in conflict with a previous study that demonstrated no significant difference in gait velocity between severity levels of DHI (32). The participants in this study were generally younger than those in the study by Whitney et al. (32) which could be one explanation for the different findings. However, different analysis methods and description of pace hamper direct comparisons. In this study, the adjusted regression analyses found that fast, but not preferred, gait velocity was significantly associated with DHI. This is in contrast to a previous study (28) and possibly due to the differences in versions of the DHI and statistical methods used. Since both studies suggest gait to influence DHI scores, results are considered not to be conflicting. The adjusted model also found significant associations between DHI and NRS_dizziness, but the lack of previous studies using this measure hampers comparison. Overall, less than half of the physical tests were significantly associated with the DHI, and the model explained only 13% of the DHI variance. These results indicate that the selected physical tests were unable to capture the current population's perception of dizziness-related handicap as measured by the DHI.

There was a trend for worse scores with increasing severity level of the DHI in the psychological measures. However, since ACQ and BSQ scores were within normal range (equal to a community sample) (54) across all DHI severity levels, the symptoms of panic and anxiety were of limited importance in this study population. Also, the MI-A (avoidance) and HADS (psychological distress) scores increased with each DHI level, in line with the previous studies on populations with dizziness (30, 55, 56). Although only participants in the severe DHI level presented with abnormal scores for MI-A and HADS, [MI-A 2.30 points (cutoff value; 1.65 points (54), HADS 15.5 (cutoff value; 12 points (57)], the regression model including the psychological outcomes (Group 2) explained a relatively large part of the DHI variance (43%), with half of the selected measures being significantly associated with the DHI. This indicates that psychological outcomes may play an important role when dizziness-related handicap increase. This is in line with previous studies that have documented avoidance behavior in these patients (5, 30, 55), and that avoidance correlates with DHI scores (30). The same applies to HADS and association with DHI scores (56, 58) and was not surprising given the reported link between vestibular diseases and psychological complaints (18).

The outcomes in Group 3 (fatigue, dizziness severity, and QoL) also presented with worse scores with increasing DHI severity levels. It was surprising that the participants in the severe DHI level had CFQ scores similar to patients with chronic fatigue syndrome (mean; 24 points) (59), and that all three current levels of the DHI presented with severe dizziness according to VSS-SF (from 12.53 to 27.0, mean 20.58; cutoff 12 points) (60). The adjusted regression model showed significant associations between DHI and VSS-SF, in addition to EQ-VAS, in line with previous studies (29, 30, 38), whereas CFQ was not associated with DHI scores. This was somewhat surprising and may be explained by the fact that none of the items in DHI directly address questions related to fatigue.

The final adjusted model including significant measures from the previous models indicated that the self-reported outcomes covering avoidance (MI-A), anxiety, and depression (HADS) in addition to dizziness severity (VSS-SF) and QoL (EQ-VAS) and had a strong relationship with the DHI, whereas the physical tests did not. The model explained 56% of the variance in the DHI scores which was considered to be rather high, and in line with another study (30) who found that a model of different psychological outcomes could explain 62.7% of the variance in the DHI scores. Another study evaluating the relationship between demographic characteristics, mental health and dizziness-related characteristics also found significant associations between DHI and several outcomes which explained 63% of the variance (29). As far as we are aware of, this study is the only study to use multivariate regression analyses including both physical tests and self-reported outcomes in a stepwise backward model that at each step gradually eliminates variables from the regression model to find a reduced model that best explains the data. This represents a rather novel approach. As none of the physical tests were significant in the final model, it could indicate that the DHI is unable to capture problems associated with everyday physical activities using gait as an example. Diverging results are reported by others concerning such associations (27, 28, 32, 61). Studies using different static balance and functional tests have found moderate to strong correlations with the DHI, like for instance, single-leg stance and Dynamic Gait Index (27, 32, 61), whereas other outcomes such as Romberg test and single-leg stand test had weak or no correlation (27, 32). Whether other physical measures would have rendered the significant associations to remain in this study is, however, unclear. From our point of view, the DHI seems to be useful in collecting self-reported information concerning psychological problems, dizziness severity, and QoL. However, as people with dizziness often present with a variety of complaints, it is necessary to use a combination of tests and measures in the assessment of individual patients with persistent dizziness.

The study has several limitations. As data were collected in relation to an ongoing RCT, power calculations were not performed for this study. The study has a cross-sectional design, and data from this study do not allow for inferring conclusions regarding direction and causality. Even though there are risks of confounders that may influence the association in cross-sectional studies, this was not likely, since this study tested for assumptions for regression analyses, and no sign of multicollinearity was found.

A weakness of this study could conceivably be that diagnoses are not included as a confounder in the association analysis. However, we recruited persons with prolonged dizziness (average 24 months) without paying attention to diagnoses as they have shown to be of less importance for function when dizziness persists (13). We rather aimed to explore what functional challenges the participants had and if these problems influenced scores on the DHI. Another possible limitation is the use of three severity levels of the DHI (32), which is neither based on evidence nor consensus. However, we opted to use the categorization due to the clinical impression that scoring in the severe DHI level could be an indication for directing special attention toward these patients. Jacobson and Newman (24) also included subscales (physical, functional, and emotional) of the DHI. However, the validity of these subscales has been questioned (62) and the use of the total DHI score has been recommended (31, 35, 36, 38, 63), and thus, only the relationship between the total DHI and selected outcomes was examined in this study.

Strengths of the study include the inclusion of a relatively large population with persistent dizziness. Data were collected systematically following a published protocol (33) and trained testers were used. It is further a strength that the current age and sex distribution equal previous studies on populations with vestibular populations (13, 29, 30), and patients attending an oto-neurology clinic due to dizziness (30, 32), thereby increasing generalizability.

## Conclusion

The use of a regression analysis including groups of physical tests and self-reported measures, to reveal the most important factors to explain the variance in DHI scores, represents a new approach in this field. The findings indicate that DHI seems to cover self-reported aspects such as avoidance, behavior, psychological distress, dizziness severity, and QoL. However, the relationship between DHI and physical tests was not established. From this study, it seems that DHI should be complemented with physical tests to establish a more complete picture of the patients‘ complaints. However, further studies are needed to establish which physical test will be the most appropriate to use together with DHI.

## Data Availability Statement

The raw data supporting the conclusions of this article will be made available by the authors, without undue reservation.

## Ethics Statement

The studies involving human participants were reviewed and approved by the Regional Committee for Medical Research Ethics. The patients/participants provided their written informed consent to participate in this study.

## Author Contributions

The primary investigator for the study was LK. She developed the study in collaboration with LM, SM, KW, SHN, RC, and BJ-K. LK was responsible for the screening and testing of participants, with assistance from LM. LK completed the statistical analysis in collaboration with EB. LK drafted the manuscript with contributions from all authors with critical revision. All authors have read and approved the final manuscript.

## Funding

This study was funded by the Norwegian Fund for Postgraduate Training in Physiotherapy. The funding body had no active part in planning and conducting the study.

## Conflict of Interest

The authors declare that the research was conducted in the absence of any commercial or financial relationships that could be construed as a potential conflict of interest.

## Publisher's Note

All claims expressed in this article are solely those of the authors and do not necessarily represent those of their affiliated organizations, or those of the publisher, the editors and the reviewers. Any product that may be evaluated in this article, or claim that may be made by its manufacturer, is not guaranteed or endorsed by the publisher.

## References

[1] Neuhauser HK Lempert T. Vertigo: epidemiologic aspects. Seminar Neurol. (2009) 29:473–81. 10.1055/s-0029-1241043 19834858

[2] Bisdorff A Bosser G Gueguen R Perrin P. The epidemiology of vertigo, dizziness, and unsteadiness and its links to co-morbidities. Front Neurol. (2013) 4:29. 10.3389/fneur.2013.00029 23526567 PMC3605504

[3] Norway S. Levekårsundersøkelse Statistisk sentralbyrå 2019. (2019). Available from: https://www.ssb.no/statbank/table/04432/tableViewLayout1/ (accessed March 24, 2022).

[4] Baloh RW. Clinical practice. Vestibular neuritis. N Engl J Med. (2003) 348:1027–32. 10.1056/NEJMcp021154 12637613

[5] Heinrichs N Edler C Eskens S Mielczarek MM Moschner C. Predicting continued dizziness after an acute peripheral vestibular disorder. Psychosom Med. (2007) 69:700–7. 10.1097/PSY.0b013e318151a4dd 17766688

[6] Godemann F Siefert K Hantschke-Bruggemann M Neu P Seidl R Strohle A. What accounts for vertigo one year after neuritis vestibularis - anxiety or a dysfunctional vestibular organ? J Psychiatr Res. (2005) 39:529–34. 10.1016/j.jpsychires.2004.12.006 15992562

[7] Bisdorff A Von Brevern M Lempert T Newman-Toker DE. Classification of vestibular symptoms: towards an international classification of vestibular disorders. J Vestib Res. (2009) 19:1–13. 10.3233/VES-2009-0343 19893191

[8] Schniepp R Wuehr M Huth S Pradhan C Brandt T Jahn K. Gait characteristics of patients with phobic postural vertigo: effects of fear of falling, attention, and visual input. J Neurol. (2014) 261:738–46. 10.1007/s00415-014-7259-1 24519356

[9] Coelho Junior AN Gazzola JM Gabilan YP Mazzetti KR Perracini MR Gananca FF. Head and shoulder alignment among patients with unilateral vestibular hypofunction. Rev Bras Fisioter. (2010) 14:330–6. 10.1590/S1413-35552010005000022 20949233

[10] Wilhelmsen K Kvale A. Examination and treatment of patients with unilateral vestibular damage, with focus on the musculoskeletal system: a case series. Phys Ther. (2014) 94:1024–33. 10.2522/ptj.20130070 24557651

[11] Allum JH Adkin AL Carpenter MG Held-Ziolkowska M Honegger F Pierchala K. Trunk sway measures of postural stability during clinical balance tests: effects of a unilateral vestibular deficit. GaitPosture. (2001) 14:227–37. 10.1016/S0966-6362(01)00132-1 11600326

[12] Horlings CG Kung UM Bloem BR Honegger F Van AN Van Engelen BG. Identifying deficits in balance control following vestibular or proprioceptive loss using posturographic analysis of stance tasks. ClinNeurophysiol. (2008) 119:2338–46. 10.1016/j.clinph.2008.07.221 18782677

[13] Wilhelmsen K Ljunggren AE Goplen F Eide GE Nordahl SH. Long-term symptoms in dizzy patients examined in a university clinic. BMCEar Nose Throat Disord. (2009) 9:2. 10.1186/1472-6815-9-2 19445693 PMC2693507

[14] Kvale A Wilhelmsen K Fiske HA. Physical findings in patients with dizziness undergoing a group exercise programme. Physiother Res Int. (2008) 13:162–75. 10.1002/pri.402 18504784

[15] Eckhardt-Henn A Breuer P Thomalske C Hoffmann S Hopf H. Anxiety disorders and other psychiatric subgroups in patients complaining of dizziness. J Anxiety Disord. (2003) 17:369–88. 10.1016/S0887-6185(02)00226-8 12826087

[16] Edelman S Mahoney AE Cremer PD. Cognitive behavior therapy for chronic subjective dizziness: a randomized, controlled trial. Am J Otolaryngol. (2012) 33:395–401. 10.1016/j.amjoto.2011.10.009 22104568

[17] Holmberg J Karlberg M Harlacher U Magnusson M. Experience of handicap and anxiety in phobic postural vertigo. Acta Otolaryngol. (2005) 125:270–5. 10.1080/00016480410023001 15966696

[18] Lahmann C Henningsen P Brandt T Strupp M Jahn K Dieterich M. Psychiatric comorbidity and psychosocial impairment among patients with vertigo and dizziness. J Neurol Neurosurg Psychiatry 86:302–8. 10.1136/jnnp-2014-307601 24963122

[19] Yardley L Burgneay J Nazareth I Luxon L. Neuro-otological and psychiatric abnormalities in a community sample of people with dizziness: a blind, controlled investigation. J Neurol Neurosurg Psychiatry. (1998) 65:679–84. 10.1136/jnnp.65.5.679 9810937 PMC2170353

[20] Neuhauser HK Radtke A von Brevern M Lezius F Feldmann M Lempert T. Burden of dizziness and vertigo in the community. Arch Intern Med. (2008) 168:2118–24. 10.1001/archinte.168.19.2118 18955641

[21] Weidt S Bruehl AB Straumann D Hegemann SC Krautstrunk G Rufer M. Health-related quality of life and emotional distress in patients with dizziness: a cross-sectional approach to disentangle their relationship. BMC Health Serv Res. (2014) 14:317. 10.1186/1472-6963-14-317 25052136 PMC4119060

[22] Iglebekk W Tjell C Borenstein P. Pain and other symptoms in patients with chronic benign paroxysmal positional vertigo (BPPV). Scand J Pain. (2013) 4:233–40. 10.1016/j.sjpain.2013.06.004 29913653

[23] Fong E Li C Aslakson R Agrawal Y. Systematic review of patient-reported outcome measures in clinical vestibular research. Arch phys Med Rehabil. (2015) 96:357–65. 10.1016/j.apmr.2014.09.017 25305629 PMC4306632

[24] Jacobson GP Newman CW. The development of the dizziness handicap inventory. Arch Otolaryngol Head Neck Surg. (1990) 116:424–7. 10.1001/archotol.1990.01870040046011 2317323

[25] Yip CW Strupp M. The dizziness handicap inventory does not correlate with vestibular function tests: a prospective study. J Neurol. (2018) 265:1210–8. 10.1007/s00415-018-8834-7 29557501

[26] Patel M Arshad Q Roberts RE Ahmad H Bronstein AM. Chronic symptoms after vestibular neuritis and the high velocity vestibulo-ocular reflex. Otol Neurotol. (2016) 37:179. 10.1097/MAO.0000000000000949 26719963 PMC4712355

[27] Vereeck L Truijen S Wuyts FL Van De Heyning PH. The dizziness handicap inventory and its relationship with functional balance performance. Otol Neurotol. (2007) 28:87–93. 10.1097/01.mao.0000247821.98398.0d 17195749

[28] Zanotto D Mamuyac EM Chambers AR Nemer JS Stafford JA Agrawal SK. Dizziness handicap inventory score is highly correlated with markers of gait disturbance. Otol Neurotol. (2017) 38:1490–9. 10.1097/MAO.0000000000001586 28984811

[29] Formeister EJ Krauter R Kirk L Zhu TR Rizk HG Sharon JD. Understanding the Dizziness Handicap Inventory (DHI): a cross sectional analysis of symptom factors that contribute to DHI variance. Otol Neurotol. (2020) 41:86–93. 10.1097/MAO.0000000000002438 31644479

[30] Herdman D Norton S Pavlou M Murdin L Moss-Morris R. Vestibular deficits and psychological factors correlating to dizziness handicap and symptom severity. J Psychosom Res. (2020) 132:109969. 10.1016/j.jpsychores.2020.109969 32097770

[31] Piker EG Jacobson GP Newman CW. Assessing dizziness-related quality of life. In: Jacobson GP, Shepard NT, Barin K, Janky K, McCaslin DL, editors. Balance Function Assessment and Management. 3rd ed. San Diego, CA: Plural publishing (2019). p. 143–66.

[32] Whitney SL Wrisley DM Brown KE Furman JM. Is perception of handicap related to functional performance in persons with vestibular dysfunction. Otol Neurotol. (2004) 25:139–43. 10.1097/00129492-200403000-00010 15021773

[33] Kristiansen L Magnussen L Wilhelmsen K Mæland S Nordahl S Clendaniel R. Efficacy of intergrating vestibular rehabilitation and cognitive behaviour therapy in persons with persistent dizziness in primary care-a study protocol for a randomised controlled trial. Trials. (2019) 20:575. 10.1186/s13063-019-3660-5 31590692 PMC6781377

[34] Von Elm E Altman DG Egger M Pocock SJ Gøtzsche PC Vandenbroucke JP. The Strengthening the Reporting of Observational Studies in Epidemiology (STROBE) statement: guidelines for reporting observational studies. Ann Intern Med. (2007) 147:573–7. 10.7326/0003-4819-147-8-200710160-00010 17938396

[35] Perez N Garmendia I Garcia-Granero M Martin E Garcia-Tapia R. Factor analysis and correlation between dizziness handicap inventory and dizziness characteristics and impact on quality of life scales. Acta Otolaryngol Suppl. (2001) 545:145–54. 10.1080/000164801750388333 11677730

[36] Vereeck L Truijen S Wuyts FL Van De Heyning PH. Internal consistency and factor analysis of the Dutch version of the Dizziness Handicap Inventory. Acta Otolaryngol. (2007) 127:788–95. 10.1080/00016480601075464 17729178

[37] Kurre A van Gool CJ Bastiaenen CH Gloor-Juzi T Straumann D de Bruin ED. Translation, cross-cultural adaptation and reliability of the german version of the dizziness handicap inventory. Otol Neurotol. (2009) 30:359–67. 10.1097/MAO.0b013e3181977e09 19225437

[38] Tamber AL Wilhelmsen KT Strand LI. Measurement properties of the Dizziness Handicap Inventory by cross-sectional and longitudinal designs. Health Qual Life Outcomes. (2009) 7:101. 10.1186/1477-7525-7-101 20025754 PMC2804706

[39] Hall CD Herdman SJ. Reliability of clinical measures used to assess patients with peripheral vestibular disorders. JNeurolPhysTher. (2006) 30:74–81. 10.1097/01.NPT.0000282571.55673.ed 16796772

[40] Bohannon RW. Muscle strength: clinical and prognostic value of hand-grip dynamometry. Curr Opin Clin Nutr Metab Care. (2015) 18:465–70. 10.1097/MCO.0000000000000202 26147527

[41] Bobos P Nazari G Lu Z MacDermid JC. Measurement properties of the hand grip strength assessment: a systematic review with meta-analysis. Arch Phys Med Rehabil. (2020) 101:553–65. 10.1016/j.apmr.2019.10.183 31730754

[42] Kvåle A Ljunggren AE Johnsen TB. Examination of movement in patients with long-lasting musculoskeletal pain: reliability and validity. Physiother Res Int. (2003) 8:36–52. 10.1002/pri.270 12701464

[43] Hawker GA Mian S Kendzerska T French M. Measures of adult pain: Visual analog scale for pain (vas pain), numeric rating scale for pain (nrs pain), mcgill pain questionnaire (mpq), short-form mcgill pain questionnaire (sf-mpq), chronic pain grade scale (cpgs), short form-36 bodily pain scale (sf-36 bps), and measure of intermittent and constant osteoarthritis pain (icoap). Arthritis Care Res/. (2011) 63.S11:S240–S252. 10.1002/acr.20543 22588748

[44] Chambless DL Caputo GC Jasin SE Gracely EJ Williams C. The mobility inventory for agoraphobia. Behav Res Ther. (1985) 23:35–44. 10.1016/0005-7967(85)90140-8 3985915

[45] Chambless DL Caputo GC Bright P Galalgher R. Assessment og fear of fear in agoraphobics: the body sensations questionnaire and the agoraphobic cognitions questionnaire. J Consult Clin Psychol. (1984) 52:10–1097. 10.1037/0022-006X.52.6.1090 6520279

[46] Zigmond A Snaith R. The hospital anxiety and depression scale. Acta Psychiatr Scand. (1983) 67:361–70. 10.1111/j.1600-0447.1983.tb09716.x 6880820

[47] Piker EG Kaylie DM Garrison D Tucci DL. Hospital anxiety and depression Scale: factor structure, internal consistency and convergent validity in Patients with dizziness. Audiol Neurotol. (2015) 20:394–9. 10.1159/000438740 26460986

[48] Chalder T Berelowitz G Pawlikowska T Watts L Wessely S Wright D. Development of a fatigue scale. J Psychosom Res. (1993) 37:147–53. 10.1016/0022-3999(93)90081-P 8463991

[49] Yardley L Masson E Verschuur C Haacke N Luxon L. Symptoms, anxiety and handicap in dizzy patients: development of the vertigo symptom scale. J Psychosom Res. (1992) 36:731–41. 10.1016/0022-3999(92)90131-K 1432863

[50] Wilhelmsen K Strand LI Nordahl SH Eide GE Ljunggren AE. Psychometric properties of the vertigo symptom scale - short form. BMC Ear Nose Throat Disord. (2008) 8:2. 10.1186/1472-6815-8-2 18371190 PMC2329601

[51] Brooks R Group E. EuroQol: the current state of play. Health Policy. (1996) 37:53–72. 10.1016/0168-8510(96)00822-6 10158943

[52] Van Reenen M, Janssen, B,. EQ-5D-5L User Guide: Basic Information on How to Use the EQ-5D-5L Instrument. (2015). Available online at: http://Euroqolorg:Euroqol (accessed August 20, 2020).

[53] Hurst N Kind P Ruta D Hunter M Stubbings A. Measuring health-related quality of life in rheumatoid arthritis: validity, responsiveness and reliability of EuroQol (EQ-5D). Br J Rheumatol. (1997) 36:551–9. 10.1093/rheumatology/36.5.551 9189057

[54] Hovland A Johansen H Sjøbø T Vøllestad J Nordhus IH Pallesen S. A feasibility study on combining internet-based cognitive behaviour therapy with physical exercise as treatment for panic disorder—treatment protocol and preliminary results. Cogn Behav Ther. (2015) 44:275–87. 10.1080/16506073.2015.1022596 25785484

[55] Jacob RG Whitney SL Detweiler-Shostak G Furman JM. Vestibular rehabilitation for patients with agoraphobia and vestibular dysfunction: a pilot study. J Anxiety Disord. (2001) 15:131–46. 10.1016/S0887-6185(00)00047-5 11388356

[56] Cheng YY Kuo CH Hsieh WL Lee SD Lee WJ Chen LK. Anxiety, depression and quality of life (QoL) in patients with chronic dizziness. Arch Gerontol Geriatr. (2012) 54:131–5. 10.1016/j.archger.2011.04.007 21561671

[57] Hosaka T Awazu H Aoki T Okuyama T Yamawaki S. Screening for adjustment disorders and major depression in otolaryngology patients using the Hospital Anxiety and Depression Scale. Int J Psychiatry Clin Pract. (1999) 3:43–8. 10.3109/13651509909024758 24945066

[58] Roh KJ Kim MK Kim JH Son EJ. Role of emotional distress in prolongation of dizziness: a cross-sectional study. J Audiol Otol. (2018) 22:6. 10.7874/jao.2017.00290 29325393 PMC5784367

[59] Cella M Chalder T. Measuring fatigue in clinical and community settings. J Psychosom Res. (2010) 69:17–22. 10.1016/j.jpsychores.2009.10.007 20630259

[60] Yardley L Donovan-Hall M Smith HE Walsh BM Mullee M Bronstein AM. Effectiveness of primary care-based vestibular rehabilitation for chronic dizziness. Ann Intern Med. (2004) 141:598–605. 10.7326/0003-4819-141-8-200410190-00007 15492339

[61] Gill-Body KM Beninato M Krebs DE. Relationship among balance impairments, functional performance, and disability in people with peripheral vestibular hypofunction. Phys Ther. (2000) 80:748–58. 10.1093/ptj/80.8.748 10911413

[62] Asmundson GJ Stein MB Ireland D. A factor analytic study of the dizziness handicap inventory: does it assess phobic avoidance in vestibular referrals? J Vestib Res. (1999) 9:63–8. 10.3233/VES-1999-9108 10334018

[63] Koppelaar-van Eijsden HM Schermer TR Bruintjes TD. Measurement properties of the dizziness handicap inventory: a systematic review. Otol Neurotol. (2022) 43:e282–97. 10.1097/MAO.0000000000003448 35147600

